# Association between type of drinking water and upper gastrointestinal cancer incidence in the Linxian General Population

**DOI:** 10.1186/s12885-023-10887-2

**Published:** 2023-05-04

**Authors:** Huan Yang, Jian-bing Wang, Xiao-kun Wang, Jin-hu Fan, You-lin Qiao

**Affiliations:** 1 Department of Cancer Epidemiology, National Cancer Center/National Clinical Research Center for Cancer/Cancer Hospital, Chinese Academy of Medical Sciences and Peking Union Medical College, 17 South Pan Jia Yuan Lane, Beijing, 100021 China; 2 Department of Public Health, and Department of Endocrinology, the Children’s Hospital, Zhejiang University School of Medicine, National Clinical Research Center for Children’s Health, Hangzhou, China

**Keywords:** Drinking water, Esophageal Neoplasms, Stomach Neoplasms, Cohort Study, China

## Abstract

**Background:**

This study aimed to explore the association between drinking water source and risk of upper gastrointestinal (UGI) cancer, including esophageal cancer (EC) and gastric cancer (GC), in the Linxian General Population Nutrition Intervention Trial (NIT) cohort.

**Methods:**

In this study, we used data from the Linxian NIT cohort, which included 29,584 healthy adults aged 40 to 69 years. Subjects were enrolled in April 1986 and followed up until March 2016. Tap water drinking status and demographic characteristics were collected at baseline. Subjects who drank tap water were treated as the exposed group. Hazard ratios (HRs) and 95% confidence intervals (95% CIs) were estimated using the Cox proportional hazard model.

**Results:**

A total of 5,463 cases of UGI cancer were identified during the 30-year follow-up period. After adjusting for multiple factors, the incidence rate of UGI cancer in participants who drank tap water was significantly lower compared with individuals in the control (HR = 0.91, 95% CI: 0.86–0.97). A similar association was observed between tap water drinking and EC incidence (HR = 0.89, 95% CI: 0.82–0.97). The association between drinking tap water and risk of UGI cancer and EC incidence did not vary across the subgroup by age and gender (All *P*_*interaction*_ > 0.05). For EC incidence, an interaction effect was observed for riboflavin/niacin supplements and drinking water source (*P*_*interaction*_ = 0.03). No association was observed between drinking water source and GC incidence.

**Conclusions:**

In this prospective cohort study in Linxian, participants who drank tap water had a lower risk of EC incidence. As a source of drinking water, use of tap water may reduce the risk of EC by avoiding exposure to nitrate/nitrite. Measures should be taken to improve the quality of drinking water in high-incidence areas of EC.

**Trial registration:**

The trial is registered with ClinicalTrials.gov (NCT00342654, 21/06/2006), and the trial name is Nutrition Intervention Trials in Linxian Follow-up Study.

**Supplementary Information:**

The online version contains supplementary material available at 10.1186/s12885-023-10887-2.

## Background

Both lifestyle and environmental factors are important risk factors for the development of cancer [1, 2], and lifestyle determinants have been found to be significant predictors of environmental carcinogens in humans [3–5]. In recent years, the causal relationship between water environments and cancer epidemiology has been extensively studied on multiple scales. Studies have shown that populations in areas with high exposure to organic or inorganic pollutants in water environments may experience high cancer or non-cancer mortality [6–8]. Since 2004, certain villages in the Chinese Huai River Basin with high mortality from cancers, referred to as "cancer villages," have been widely reported by the domestic media [9]. In 2013, the Chinese Center for Disease Control and Prevention directly confirmed the association between water pollution and cancer incidence in their research on the Correlation between Cancer and the Huai River Water Environment [10]. This study demonstrated that cancer incidence increased in the 10–15 years after water pollution in the Huai River basin, and pollution levels had a dose–response relationship with cancer incidence/mortality. Additionally, pollutants in the water environment enter the human body, and their metabolites are found in human blood and tissues [10]. Drinking water is known to be an effective exposure route for carcinogenic contaminants, as any chemicals generated by human activity can and will find their way into water supplies [11]. Drinking water habits, as a lifestyle factor, may to some extent reflect pollution in the environment surrounding the drinking water source. Both biological and chemical contaminants (such as nitrate, phosphates, heavy metals, and endocrine-disrupting chemicals) in drinking water can have short- and long-term health effects [12]. Moreover, toxic chemicals ingested from the environment can be present in a variety of human biological samples, including cord blood, placental tissues, and amniotic fluid, which can be transmitted to the fetus through the placenta, leading to adverse pregnancy outcomes [13, 14].

China is a country with high incidence of esophageal (EC) and gastric cancer (GC), both of which are among the top ten most common cancers and top five leading causes of cancer deaths in the Chinese population [15]. In recent years, studies have examined the relationship between drinking water quality and the occurrence of these cancers. A previous study in Iran, a high-incidence country for EC, showed that drinking un-piped water, in addition to common risk factors such as smoking, nutrient-deficient diets, and drinking hot tea, significantly increased the risk of EC incidence [16]. High concentrations of nitrate and nitrite in water are a common characteristic among most countries with a high GC incidence, such as Chile, Costa Rica, and Japan [17]. Linxian, a rural county of Anyang, Henan Province, is one of the high-incidence regions of upper gastrointestinal (UGI) cancer in China [18]. The region's arid climate and low annual rainfall make surface water, such as mountain spring water and shallow well water, the primary water resource [19]. Carcinogens in drinking water were hypothesized to be associated with high cancer incidence, leading to a series of drinking water tests and animal cancer-inducing tests in Linxian. Investigations conducted in the 1980s showed that natural drinking water sources in Linxian contained higher levels of nitrate and nitrite [20]. Etiological studies of digestive cancer have shown that concentrated drinking water can induce esophageal neoplasms and gastric papillomas in mice [21]. The government started to improve water quality in Linxian by installing tap water from the 1970s to 1980s, but few prospective studies evaluated the protective effects of drinking tap water on decreased UGI cancer risk.

In the 1980s, a Nutrition Intervention Trial (NIT) was initiated in Linxian's general population to explore whether vitamin and mineral supplements could reduce cancer incidence and common chronic disease mortality. At baseline, all participants' use of tap water was collected, and the follow-up lasted until the present day. We assumed that UGI cancer incidence varied by the type of drinking water, and in this study, we examined the association between drinking water and the risk of UGI cancer incidence based on the 30-year follow-up data in the Linxian NIT cohort.

## Material and methods

### Study population

This prospective observational cohort study was conducted between 1986 and 2016 on healthy adults residing in Linxian. The detailed design of the NIT cohort has been previously described [22, 23]. Potential participants were eligible if they were between the ages of 40 and 69, lived in one of the communes in northern Linxian (Yaocun, Rencun, Donggang, and Hengshui), volunteered to participate in the study, and provided consent. Subjects with malignancy, regular intake of vitamin and mineral supplements, or regular use of specific medications such as the traditional drug Anti-Tumor B3 or synthetic retinoids were excluded from this study. Finally, a total of 29,584 subjects were included in the NIT study in 1986. All participants received daily supplementation based on a factorial design consisting of four factors: factor A (retinol/zinc), factor B (riboflavin/niacin), factor C (vitamin C/molybdenum), and factor D (selenium/vitamin E/beta-carotene) [21]. After a baseline survey, subjects were randomly assigned to one of eight intervention groups based on a 2^4^ fractional factorial design, which received factors ABCD, AB, AC, AD, BC, BD, CD, or placebo, respectively. With this design, half of the subjects received each of the four factors and half did not [23]. The doses of these daily supplements are shown in Supplementary Table S1. Before enrollment, each participant was asked to provided informed consent. The intervention lasted from March 1986 to May 1991. This study was approved by the institutional review boards of the Cancer Hospital Chinese Academy of Medical Sciences (CHCAMS) and the US National Cancer Institute (NCI).

### Baseline survey

All participants underwent a physical examination at local village hospitals and completed a standardized questionnaire administered by trained study personnel and research technicians. The protocol included quality control measures to ensure consistency across all data collected. The standard questionnaire was used to collect demographic and lifestyle characteristics at baseline, including age, sex, smoking, alcohol consumption, educational level, family history of UGI cancer, and dietary habits. Smoking was defined as the regular use of cigarettes or pipes for at least six months, and alcohol use was categorized as daily, weekly, monthly, yearly, or never according to the frequency of use in the past 12 months. Participants were asked whether they had tap water installed as a source of drinking water in their home, and responses were categorized as 'yes' or 'no'. To assess fresh fruit and vegetable intake, participants were asked how often they consumed fresh fruit and vegetable in the 12 months prior to the interview. To avoid seasonal effects, the frequency of fruit and vegetable consumption in winter/spring and summer/autumn was calculated separately. Responses could be 'never', 'times/day', 'times/week', 'times/month', or 'times/year' and were all converted to one unit (times/year). We also collected data on the frequency of preserved vegetable, moldy vegetable juices, and moldy staple foods consumption (times/year), given the potential presence of dietary carcinogens. Family history of UGI cancer was considered positive if the subject had one or more first-degree relatives diagnosed with esophageal cancer or gastric cancer. Trained staff measured participants' height and weight while subjects were not wearing shoes according to a standard protocol. Body mass index (BMI) was calculated as weight in kilograms divided by height in meters squared (kg/m^2^). Additionally, we collected information on the history of consumptive disease. Participants were asked whether they had ever been diagnosed with a severe disease that prevented them from doing household chores or performing labor (such as cardiovascular disease, stroke, cirrhosis, or diabetes mellitus), and the answer was recorded as 'yes' or 'no'.

### Follow-up

During the 5.25-year trial period (March 1986—May 1991), village medical staff visited all participants monthly to deliver pills, assess compliance, ascertain endpoint events, and collect diagnostic materials. In this period, cancer cases were confirmed by an International Endpoints Review Committee consisting of American and Chinese senior experts. After the trial period, local medical workers continued to visit surviving participants every three months. New cancer cases and all-cause deaths were verified by a panel of American and Chinese experts from 1991 to 1996, and by senior Chinese doctors from 1996 to 2017, using diagnostic materials such as case records, biochemical results, ultrasound, X-rays, endoscopy, surgery reports, and pathology or cytology slides.

### Statistical analysis

Participants were censored at the last known follow-up date, date of death, or the date of the study closure (March 2016), whichever came first. The primary outcomes of our study were primary EC (ICD10: C15) and GC (ICD10: C16) incidence. Participants were divided into two categories according to their tap water drinking status (do not drink tap water and drinking tap water), with no tap water-drinking as the reference. Differences in baseline demographic characteristics between different tap water drinking statuses were compared using the Chi-square test for categorical variables and t-test or nonparametric tests for continuous variables. Univariate and multivariable Cox proportional hazards regression models were used to calculate hazard ratios (HRs) and 95% confidence intervals (95% CIs) for the association between tap water drinking and the risk of UGI cancer incidence. Potential covariates in the models included age at baseline, gender, BMI, smoking, alcohol drinking, family history of UGI cancer, education level, nutrition intervention arms, communes, history of consumptive disease, and frequency of intakes of fresh fruit, fresh vegetables, preserved vegetables, moldy vegetable juice, and moldy staple foods. Subgroup analyses were conducted to evaluate whether the association varied by age at baseline, gender, and nutritional intervention arms. Cumulative incidence rates were estimated using the Kaplan–Meier method, and differences between the cumulative incidence curves were assessed using log-rank tests. Sensitivity analysis was conducted by excluding participants followed up for less than three years to assess reverse causality. Statistical analyses were performed using SPSS 23.0. All tests were two-tailed, and a *P* value of < 0.05 was considered statistically significant.

## Results

Overall, a total of 29,449 individuals were included in the final analysis. The CONSORT flow diagram for the Linxian Nutrition Intervention Trial is shown in Supplementary material Figure S1. During the 30-year follow-up, we identified 5,463 UGI cancer cases, including 3,103 EC cases and 2,360 GC cases. Table 1 shows the baseline characteristics of participants with different drinking tap water statuses. There were significant differences in age at baseline, BMI, smoking status, education level, communes, history of consumptive disease, and frequency of intakes of fresh fruit, fresh vegetable, and moldy staple foods (All *P* values < 0.05).

**Table 1:** Baseline characteristics in the Linxian General Population Nutrition Intervention Trial Cohort by drinking water type

	**Drinking tap water**				*P* value
	**Yes**	**%**	**No**	%	
**Age at baseline (years,mean ± sd)**	52.0 ± 8.5		51.9 ± 9.0		< 0.01
**Body mass index (kg/m**^**2**^**,mean ± sd)**	22.1 ± 2.5		21.9 ± 2.4		< 0.01
**Fresh vegetable(times/year), median (IQR)**	730.0(547.5–912.5)		730.0(547.5–912.5)		< 0.01
**Fresh fruit(times/year), median (IQR)**	6.0(3.0–18.0)		6.0(2.0–12.0)		< 0.01
**Gender**					0.55
Female	4052	55.8	12278	55.4	
Male	3216	44.2	9903	44.6	
**Commune**					< 0.01
Yaocun	3086	42.5	6833	30.8	
Rencun	378	5.2	5549	25.0	
Donggang	595	8.2	5652	25.5	
Hengshui	3209	44.2	4147	18.7	
**Smoking**					0.02
No	5164	71.1	15449	69.6	
Yes	2104	28.9	6732	30.4	
**Drinking**					0.09
No	5508	75.8	17027	76.8	
Yes	1760	24.2	5153	23.2	
**Education level**					< 0.01
Non	2736	37.6	9076	40.9	
< Primary education	2262	31.1	6922	31.2	
≥ Primary education	1551	21.3	4303	19.4	
unknown	719	9.9	1880	8.5	
**Family history of UGI cancer**					0.43
No	4965	68.3	15041	67.8	
Yes	2303	31.7	7140	32.2	
**History of consumptive disease**					0.04
No	6641	91.4	20011	90.2	
Yes	627	8.6	2170	9.8	
**Factor A**					0.98
No	3630	49.9	11082	50.0	
Yes	3638	50.1	11099	50.0	
**Factor B**					0.47
No	3610	49.7	11126	50.2	
Yes	3658	50.3	11055	49.8	
**Factor C**					0.50
No	3605	49.6	11103	50.1	
Yes	3663	50.4	11078	49.9	
**Factor D**					0.26
No	3675	50.6	11045	49.8	
Yes	3593	49.4	11136	50.2	
**Preserved vegetable consumption**					0.864
**Never**	6601	90.8	20160	90.9	
**≥ 1 time/year**	667	9.2	2021	9.1	
**Moldy staple foods consumption**					< 0.01
**Never**	6063	83.4	18083	81.5	
**≥ 1 time/year**	1205	16.6	4098	19.5	
**Moldy vegetable juice consumption**					0.854
**Never**	7236	99.6	22087	99.6	
**≥ 1 time/year**	32	0.4	94	0.4	

**Fig. 1:**
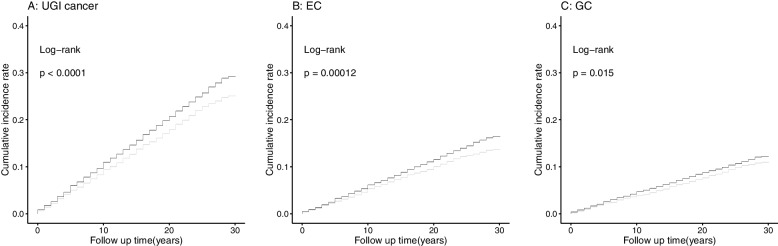
Cumulative incidence curves of total UGI cancer, EC, and GC by type of drinking water.  : non tap water group;  : tap water group

Table 2 presents the associations between drinking tap water and the risk of UGI cancer incidence. After adjusting for age at baseline and gender, the risk of UGI cancer and EC incidence in participants who drank tap water was significantly decreased by 14, 16, and 12%, respectively (HR_UGI cancer_ = 0.86, 95%CI:0.81–0.92; HR_EC_ = 0.84, 95%CI: 0.77–0.92; HR_GC_ = 0.88, 95%CI:0.80–0.98). The difference was still significant after controlling for potential confounders, including age at baseline, gender, BMI, smoking, alcohol drinking, family history of UGI cancer, education level, nutrition intervention arms, communes, history of consumptive disease, and frequency of intakes of fresh fruit, fresh vegetable, preserved vegetable, moldy vegetable juice, and moldy staple foods (HR_UGI cancer_ = 0.91, 95%CI:0.86–0.97; HR_EC_ = 0.89, 95%CI: 0.82–0.97). No association between drinking tap water and the risk of GC incidence was observed in the multivariable model (HR_GC_ = 0.94, 95%CI: 0.85–1.03). Cumulative incidence curves of total UGI cancer, EC, and GC by types of drinking water are presented in Fig. 1.

**Table 2:** HRs and 95% CIs for the associations between drinking tap water and risk of UGI cancer incidence

Cancer incidence	Drinking tap water	
	**No**	**Yes**
**UGI cancer**		
No. of cases	4249	1214
Crude HR (95%CI)	1.00	**0.86(0.81–0.92)**
Age and gender adjusted HR (95%CI)	1.00	**0.86(0.81–0.92)**
Multivariable adjusted HR (95%CI)^a^	1.00	**0.88(0.83–0.94)**
**EC**		
No. of cases	2425	678
Crude HR (95%CI)	1.00	**0.85(0.78–0.92)**
Age and gender adjusted HR (95%CI)	1.00	**0.84(0.77–0.92)**
Multivariable adjusted HR (95%CI)^a^	1.00	**0.86(0.79–0.94)**
**GC**		
No. of cases	1824	536
Crude HR (95%CI)	1.00	**0.89(0.81–0.98)**
Age and gender adjusted HR (95%CI)	1.00	**0.88(0.80–0.98)**
Multivariable adjusted HR (95%CI)^a^	1.00	0.91(0.83–1.00)

*Abbreviations*: *HR* hazard ratio, *95% CI* 95% confidence interval, *UGI* upper gastrointestinal, *EC* esophageal cancer, *GC* Gastric cancer

Bold text indicates statistical significance (*P* < 0.05)

^a^Adjusted for age at baseline, gender, BMI, smoking, alcohol drinking, family history of UGI cancer, education level, nutrition intervention arms, communes, history of consumptive disease, and frequency intakes of fresh fruit, fresh vegetable, preserved vegetable, moldy vegetable juice, and moldy staple foods

After stratification by age at baseline and gender, the associations between drinking tap water and the risk of UGI cancer incidence were significant among younger (< 55 years) participants (HR = 0.90, 95%CI:0.83–0.98) and males (HR = 0.89, 95%CI: 0.81–0.98), while for both males and females, subjects who drank tap water had 12% (HR = 0.88, 95%CI: 0.77–0.99) and 13% (HR = 0.87, 95%CI: 0.78–0.98) lower risk of EC incidence, respectively. The association between drinking tap water and the risk of UGI cancer and EC incidence did not vary by age and gender (All P _interaction_ > 0.05) (Table 3).

**Table 3:**
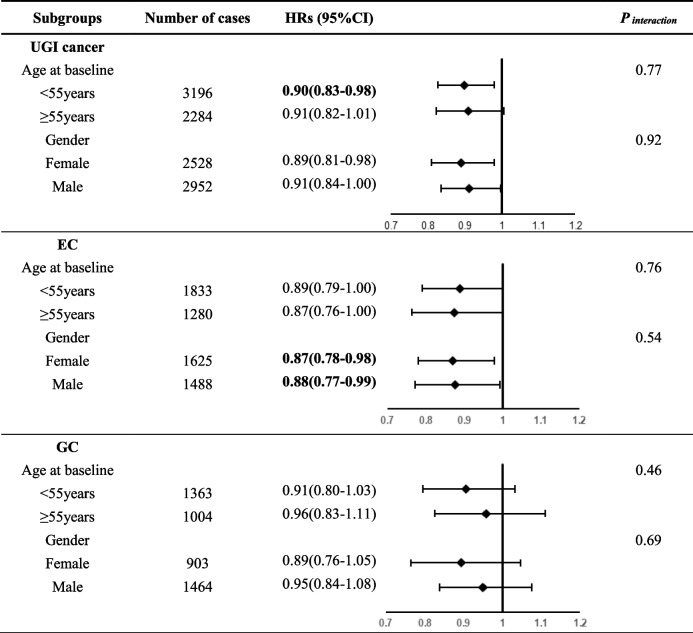
Subgroup analyses for drinking tap water and risk of UGI cancer incidence by age and gender^a^

*Abbreviations*: *HR* Hazard ratio, *95% CI* 95% Confidence interval, *UGI* Upper gastrointestinal, *EC* Esophageal cancer, *GC* Gastric cancer

Bold text indicates statistical significance (*P* < 0.05)

^a^Adjusted for age at baseline, gender, BMI, smoking, alcohol drinking, family history of UGI cancer, education level, nutrition intervention arms, communes, history of consumptive disease, and frequency intakes of fresh fruit, fresh vegetable, preserved vegetable, moldy vegetable juice, and moldy staple foods

Table 4 presents the risk of UGI cancer incidence after stratification by nutrition intervention. Among subjects who did not receive Factor B, the incidence of EC was significantly lower in the tap water group (HR_EC_ = 0.82, 95%CI: 0.73–0.93). The association between drinking tap water and the risk of EC incidence was different for riboflavin and niacin (Factor B) supplementation (P _interaction_ = 0.03). No interaction effects between drinking tap water and other nutrient supplements were observed for the risk of UGI cancer or GC incidence.

**Table 4:**
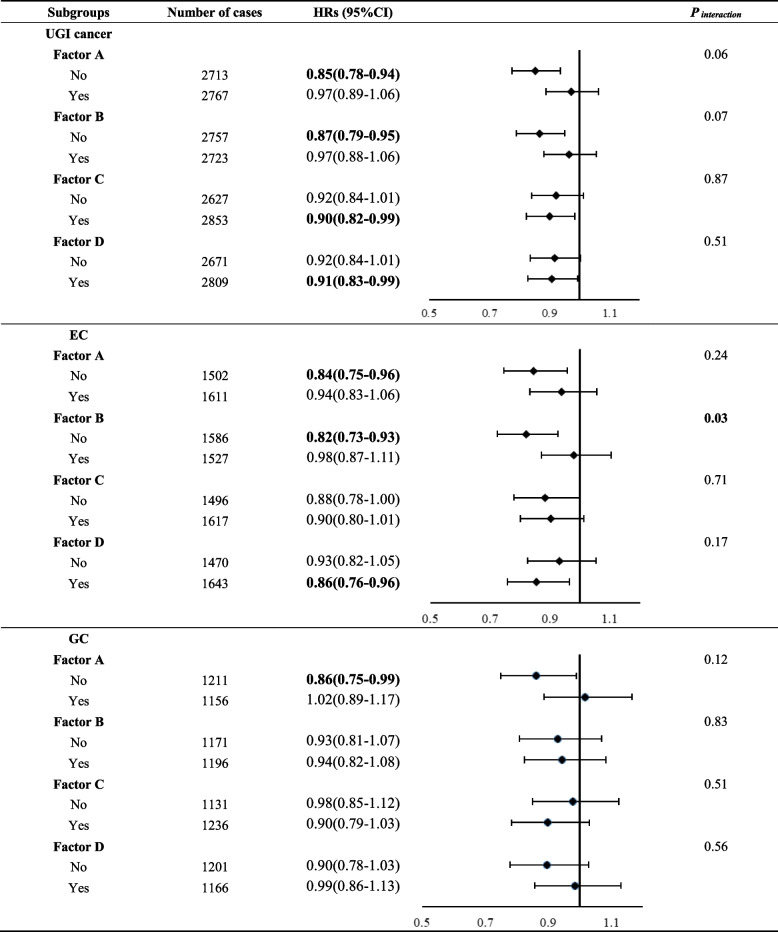
Subgroup analyses by nutrition intervention for the association between drinking tap water and risk of UGI cancer incidence^a^

*Abbreviations*: *HR* Hazard ratio, *95% CI* 95% Confidence interval, *UGI* Upper gastrointestinal, *EC* Esophageal cancer, *GC* Gastric cancer

Bold text indicates statistical significance (*P*  < 0.05)

^a^Adjusted for age at baseline, gender, BMI, smoking, alcohol drinking, family history of UGI cancer, education level, nutrition intervention arms, communes, history of consumptive disease, and frequency intakes of fresh fruit, fresh vegetable, preserved vegetable, moldy vegetable juice, and moldy staple foods

Sensitivity analysis was performed to assess reverse causality. After excluding participants with less than three years of follow-up, our results did not materially change, indicating the robustness of the study's analysis (Table 5).

**Table 5:** Sensitivity analysis by excluding individuals with less than 3 years of follow-up for the associations between drinking tap water and risk of UGI cancer incidence

Cancer incidence	Drinking tap water	
	**No**	**Yes**
**UGI cancer**		
No. of cases	3438	981
Crude HR (95%CI)	1	**0.86(0.80–0.92)**
Age and gender adjusted HR (95%CI)	1	**0.86(0.80–0.92)**
Multivariable adjusted HR (95%CI)^a^	1	**0.92(0.86–0.98)**
**EC**		
No. of cases	1983	551
Crude HR (95%CI)	1	**0.84(0.76–0.92)**
Age and gender adjusted HR (95%CI)	1	**0.83(0.76–0.91)**
Multivariable adjusted HR (95%CI)^a^	1	**0.88(0.81–0.96)**
**GC**		
No. of cases	1455	430
Crude HR (95%CI)	1	**0.89(0.80–0.99)**
Age and gender adjusted HR (95%CI)	1	**0.89(0.80–0.99)**
Multivariable adjusted HR (95%CI)^a^	1	0.96(0.87–1.07)

Abbreviations: *HR* Hazard ratio, *95% CI* 95% Confidence interval, *UGI* Upper gastrointestinal, *EC* Esophageal cancer, *GC* Gastric cancer

Bold text indicates statistical significance (*P* < 0.05)

^a^Adjusted for age at baseline, gender, BMI, smoking, alcohol drinking, family history of UGI cancer, education level, nutrition intervention arms, communes, history of consumptive disease, and frequency intakes of fresh fruit, fresh vegetable, preserved vegetable, moldy vegetable juice, and moldy staple foods

## Discussion

In this prospective cohort study, we observed that drinking tap water was associated with a reduced risk of upper gastrointestinal (UGI) cancer incidence, particularly esophageal cancer (EC). For EC incidence, we noted an interaction effect between riboflavin/niacin supplements and the type of drinking water. However, we did not find any associations with gastric cancer (GC) in all subjects or any subgroup.

The quality of drinking water is essential for human health. Despite improvements in socio-economic levels in recent decades, access to an improved water source remains a significant issue [24]. Assessing potential links between drinking water and cancer could help identify harmful chemicals that appear in water supplies at concentrations sufficient to pose a substantial attributable cancer risk. Many studies have shown the relationship between drinking water quality and the risk of breast, kidney, bladder, and colorectal cancers [21, 25–27]. However, few studies have explored the association between drinking water and UGI cancer risk. In this study, we found that participants who drank tap water had a significantly lower risk of EC incidence than those who did not drink tap water. This finding is consistent with a previous study conducted in Iran, which is also a high-incidence country for EC [12]. The association between drinking water and cancer is reportedly due to the contamination of water with chemical pollutants such as nitrite and nitrosamines [28]. Nitrosamines are a group of environmental pollutants that are mutagenic, teratogenic, and carcinogenic [29]. Darvishmotevalli M et al. investigated the carcinogenic risks related to nitrate exposure in drinking water in Iran based on a cross-sectional study [30]. The findings demonstrated that most provinces in Iran had impermissible levels of nitrate in drinking water supplies, and the carcinogen risk values of nitrate exposure through drinking water were 0.001% (Risk level for assessing the carcinogen risk was 10 − 5 [1 per 100,000 persons]). This study also indicated an association between gastrointestinal cancer and nitrate exposure through drinking water [30, 31]. In addition to direct exposure, precursors like nitrates, nitrites, and secondary and tertiary amines are potential sources of nitrosamines in vivo [32].

Before our study, researchers found that the levels of ammonia nitrogen, nitrate, and nitrite in drinking water in Linxian were significantly higher than those in other low-incidence areas [33]. Additionally, the urine nitrate and nitrite content of residents in Linxian were significantly higher than those of the control population from low-incidence areas [33]. To explore the association between drinking water in Linxian and UGI cancer, researchers concentrated well water ten times as drinking water for rats and induced EC and gastric cardia cancer [33]. Since the early 1980s, the villages in Linxian have started to improve water quality. The government mainly replaced mountain spring water, shallow well water, and canal water with deep well water, as well as replacing river water and stream water with mountain spring water [21]. However, some villages with mountain spring water did not change the water source as they did not need to invest. Residents who did not install tap water in their homes usually continued to use shallow well water due to their previous drinking habits [34]. In Linxian, most organic fertilizer comes from farmyard manure, which is the source of contamination of amines and amides. In the 1980s, the management of farmyard manure did not gain enough prominence, and compost heaps, toilets, and pig pens persisted within 30 m of the water source [34]. This could increase intakes of nitrite and amines from drinking water sources. Based on previous research on the etiology of esophageal cancer (EC), improving the quality of drinking water is considered the most effective way to reduce the incidence of EC. Our study found that after controlling for other risk factors that affect the incidence of EC, drinking tap water was associated with an 11% decreased risk of EC incidence, which supports the hypothesis that water sources can reduce the risk of EC.

The influence of drinking water quality on the incidence of upper gastrointestinal (UGI) cancer may vary depending on nutrient intake. Deficiency concentrations of trace elements also play an important role in the medical geology of esophageal cancer (EC). Some metals, when consumed through food and water, are involved in various physiological processes [35]. Keshavarzi et al. compared the concentrations of trace elements in high- and low-incidence areas of EC and found that the average concentrations of Sb, Se, Sr, and Zn in the high-incidence areas of EC were 12.2, 8.9, 3,437.3, and 30.3 µg/L, respectively, while in the low-incidence areas, they were only 4.1, 2.3, 1,102.3, and 83.3 µg/L, respectively [12]. Analysis of EC rates in high- and low-incidence areas of China has shown an inverse association between EC mortality rate and the contents of selenium in the soil [36], which may be related to the inhibitory effect of this element on indigenous nitrosamine compound production. Deficiency of trace elements in the soil has led to reductions in their concentrations in crops [37]. Under conditions of limited trace element intake from diet, the carcinogenesis of esophageal epithelium may be affected. In addition, the antioxidant effect helps counteract carcinogens, including nitrosamine and aflatoxin [38]. Fresh fruits and vegetables are good sources of dietary antioxidants [39]. The existing dietary review survey results revealed that the diet composition in Linxian was monotonous, had obvious seasonal characteristics, and the average levels of various micronutrients and fats in the diet did not meet the nutritional requirements [40]. Therefore, high levels of carcinogens and low trace minerals in natural water sources, combined with a lack of antioxidant intake, may increase the risk of EC. The combined effects of antioxidant deficiency and nitroso compound intake have been supported by animal experiments. Pan et al. evaluated the effects of N-nitroso methylbenzylamine (NMBA) and riboflavin (RBF) deficiency individually or in combination on esophageal tumorigenesis in rats, and the results suggest that RBF deficiency causes chronic inflammation-associated genomic instability that contributes to NMBA-induced esophageal tumorigenesis [41]. Odeleye et al. investigated the contribution of retroviral immunosuppression to esophageal cancer induced by the carcinogen N-nitroso methylbenzylamine (NMBzA) and the effect of increased levels of dietary vitamin E on induced carcinogenesis. They found that vitamin E could play an antioxidant role that reduced the incidence of ECs in both immunocompromised and non-immunocompromised animals [42]. In our study, the difference in UGI cancer and EC incidence between subjects who received Factor A or Factor B was not significant. This could be explained by the anti-cancer properties of nutrients. Among individuals who received nutrition supplements but did not drink tap water, the adverse effects of carcinogens in drinking water are partially offset by nutrients, resulting in a lower incidence of esophageal cancer. This finding could also support the combined effects of antioxidant deficiency and nitroso compounds intake on the risk of EC. Tap water is potable water supplied to a tap inside the household or workplace that has been treated to remove or reduce contaminants to levels where they are not expected to cause any health effects. Therefore, in areas with severe water pollution, choosing tap water as the drinking water source could effectively prevent adverse effects on human health caused by water pollution.

In our study, we did not find an association between drinking tap water and the risk of GC incidence. Limited studies are available on the relationship between drinking water with nitrate/nitrite content and the risk of GC. However, a meta-analysis conducted by Song P et al. reported significant associations of dietary intakes of nitrates and nitrites with GC observed in case–control studies, while cohort studies still showed a slight trend [43]. In some previous studies, high or moderate consumption of nitrates was even found to have a protective effect on GC [44]. Most of these studies speculated that the possible explanation could be the contribution of fruits and vegetables containing nutrients that inhibit N-nitrosation [43, 44]. During digestion in the stomach, nutrients from food are released, which can inhibit endogenous nitrosation and hinder the formation of nitrosation compounds. The function of the esophagus is to transport food and fluids from the mouth to the stomach rather than digest or absorb nutrients. Concentrations of nutrients in different subsites of the upper digestive tract may partly explain the different effect of drinking water on health status.

Our study has several strengths, including a large study population, a prospective design, a long follow-up duration, and a low rate of loss to follow-up (less than 10%). However, several limitations need to be considered. Firstly, we only conducted a one-time baseline investigation, and changes in drinking water source were not repeatedly measured. Secondly, some unmeasured factors, such as passive smoking, physical activity, smoking and alcohol drinking frequency, and socioeconomic status, could inevitably lead to residual confounding. Thirdly, since this is an observational study rather than an interventional study, contamination between the tap water group and non-tap water group could not be strictly controlled. Finally, we did not measure the levels of nitrite and trace elements in drinking water, and thus the cause of the association between drinking water source and UGI cancer risk could not be inferred.

## Conclusions

In summary, our study found that individuals who drank tap water in Linxian had a lower risk of EC incidence compared to those who did not drink tap water. This finding is consistent with previous studies on the etiology of EC, which suggest that the use of tap water as a source of drinking water can reduce the risk of esophageal cancer by avoiding nitrate/nitrite exposure. Therefore, appropriate measures should be taken to improve the quality of drinking water in high-incidence areas of EC. Furthermore, the consumption of dietary antioxidants is recommended as a protective measure.

## Supplementary Information

**Additional file 1:** **Table S1.** Doses for daily multivitamins and minerals supplementation.** Figure S1.** Consort flow diagram of the linxian general population trial.

## Data Availability

The datasets used and/or analysed during the current study are available from the corresponding author on reasonable request.
