# Supplementary material for: Association between type of drinking water and upper gastrointestinal cancer incidence in the Linxian General Population
Source: BMC Cancer. 2023 May 4;23:397. doi: 10.1186/s12885-023-10887-2 (PMC10158328; doi:10.1186/s12885-023-10887-2)
Supplement: Supplementary file 1 — Additional file 1: Table S1. Doses for daily multivitamins and minerals supplementation. Figure S1. Consort flow diagram of the linxian general population trial. [file 12885_2023_10887_MOESM1_ESM.docx]

***Supplementary material***

**Table S1.** Doses for daily multivitamins and minerals supplementation

| **Factor** | **Type of vitamin and mineral** | **Dose** |
| --- | --- | --- |
| Factor A | vitamin A | 5000 IU/d |
|  | zinc | 22.5 mg/d |
| Factor B | riboflavin | 3.2 mg/d |
|  | niacin | 40 mg/d |
| Factor C | ascorbic acid | 120 mg/d |
|  | molybdenum | 30 μg/d |
| Factor D | selenium | 50 mg/d |
|  | vitamin E | 30 mg/d |
|  | beta-carotene | 15 mg/d |

**Figure S1.** Consort Flow Diagram of the Linxian General Population Trial

Assessed for eligibility

(n=43,956)

No meeting inclusion criteria(n=1,499)

Refused to participate (n=7,992)

Difficult to be regularly followed up (n=4,182)

30,283 randomly allocated to one of eight arms

Death before start of intervention (n=153)

Cytology exclusion (n=54)

Self-reported cancer at screening (n=238)

Cancer diagnosed before start of intervention (n=254)

29,584 received treatment

(range: 3,687-3,706 per arm)

Excluded participants with missing values of included exposure (n=135)

29,449 were included in this analysis
